# “WE NEED TO BE AT THE TABLE” COLLABORATION WITH OLDER ADULTS WITH EXPERIENCES OF HOMELESSNESS

**DOI:** 10.1093/geroni/igae098.0949

**Published:** 2024-12-31

**Authors:** Sarah Canham, Rachel Weldrick, Anne Cartledge, Hilary Chapple, Chris Danielsen, Dorothy Kestle, Samantha Teichman

**Affiliations:** University of Utah, Salt Lake City, Utah, United States; McMaster University, Hamilton, Ontario, Canada; Aging In The Right Place Partnership, Calgary, Alberta, Canada; Aging In The Right Place Partnership, Calgary, Ontario, Canada; Aging In The Right Place Partnership, Vancouver, British Columbia, Canada; Aging In The Right Place Partnership, Vancouver, British Columbia, Canada; Simon Fraser University, Vancouver, British Columbia, Canada

## Abstract

Increasingly recognized as an asset to research endeavors, persons with lived experience (LE) offer key insights into the impact of research processes and outcomes with those most affected by social or medical conditions. Additionally, LE advisors and co-researchers are uniquely positioned to inform knowledge mobilization and community engagement efforts that complement research activities and disseminate findings to diverse knowledge users. This presentation will share an example of a LE advisory group that collaborated in conceptualizing and implementing a knowledge mobilization project on aging and homelessness within three Canadian cities (Vancouver, Calgary, and Montreal). Following the establishment of the advisory group, priorities and objectives were determined, resulting in community engagement initiatives to disrupt discrimination toward older adults with experiences of homelessness. We will present lessons learned from this project, including the necessity of 1) digital supports to enable inclusion of advisors, 2) honoraria for advisors’ time and contributions, 3) scheduling regular meeting days and times, and 4) dedicating meeting time for personal updates. Insights from this collaborative project are widely applicable across disciplines. This model offers a blueprint for other research teams aiming to enhance the impact of research and knowledge mobilization efforts and build capacity among LE communities.

